# Attempted and Successful Compensation in Preclinical and Early Manifest Neurodegeneration – A Review of Task fMRI Studies

**DOI:** 10.3389/fpsyt.2014.00132

**Published:** 2014-09-29

**Authors:** Elisa Scheller, Lora Minkova, Mathias Leitner, Stefan Klöppel

**Affiliations:** ^1^Section of Gerontopsychiatry and Neuropsychology, Department of Psychiatry and Psychotherapy, University Medical Center Freiburg, Freiburg, Germany; ^2^Freiburg Brain Imaging Center (FBI), University Medical Center Freiburg, Freiburg, Germany; ^3^Laboratory for Biological and Personality Psychology, Department of Psychology, University of Freiburg, Freiburg, Germany; ^4^Department of Neurology, University Medical Center Freiburg, Freiburg, Germany

**Keywords:** compensation, functional magnetic resonance imaging, Huntington’s disease, amnestic mild cognitive impairment, healthy aging

## Abstract

Several models of neural compensation in healthy aging have been suggested to explain brain activity that aids to sustain cognitive function. Applying recently suggested criteria of “attempted” and “successful” compensation, we reviewed existing literature on compensatory mechanisms in preclinical Huntington’s disease (HD) and amnestic mild cognitive impairment (aMCI). Both disorders constitute early stages of neurodegeneration ideal for examining compensatory mechanisms and developing targeted interventions. We strived to clarify whether compensation criteria derived from healthy aging populations can be applied to early neurodegeneration. To concentrate on the close coupling of cognitive performance and brain activity, we exclusively addressed task fMRI studies. First, we found evidence for parallels in compensatory mechanisms between healthy aging and neurodegenerative disease. Several studies fulfilled criteria of attempted compensation, while reports of successful compensation were largely absent, which made it difficult to conclude on. Second, comparing working memory studies in preclinical HD and aMCI, we identified similar compensatory patterns across neurodegenerative disorders in lateral and medial prefrontal cortex. Such patterns included an inverted *U*-shaped relationship of neurodegeneration and compensatory activity spanning from preclinical to manifest disease. Due to the lack of studies systematically targeting all criteria of compensation, we propose an exemplary study design, including the manipulation of compensating brain areas by brain stimulation. Furthermore, we delineate the benefits of targeted interventions by non-invasive brain stimulation, as well as of unspecific interventions such as physical activity or cognitive training. Unambiguously detecting compensation in early neurodegenerative disease will help tailor interventions aiming at sustained overall functioning and delayed clinical disease onset.

## Introduction

Two decades ago, first accounts of compensatory brain activity in healthy aging emerged in the neuroimaging literature, when an intriguing hyperactivity of several brain areas in the elderly compared to young participants was identified (1, 2). Since then, numerous studies across various cognitive domains have been conducted to understand the nature of such hyperactivity. One of the most important questions in this regard pertains to the interpretation of over-recruitment of brain areas as “true” compensation versus a sign of inefficiency due to pathology-related neural decline (3). Several theoretical frameworks have been postulated to explain adaptive processes in terms of compensation in aging and disease.

In the current review, we will first discuss existing models of compensation predominantly emerging from cognitive neuroscience of aging. Here, we will focus on a recent compensation model by Dennis and Cabeza (4), postulating explicit criteria of compensation. Then, we will compare evidence from preclinical and early manifest Huntington’s disease (HD) and amnestic mild cognitive impairment (aMCI) patients to determine if compensation in neurodegeneration follows principles similar to compensation in healthy aging. Furthermore, we will also collate potential compensatory mechanisms across neurodegenerative disorders. Based on the reviewed evidence, we will discuss implication for targeted interventions to sustain compensation.

### Theories on compensatory mechanisms in aging

In a recent review, Barulli and Stern (5) provide a comprehensive overview of compensation-related theories and their application scope not only in healthy aging, but also in instances of brain damage in general. They conclude that current theories of compensation do not contradict each other, but constitute closely related concepts, each emphasizing different aspects of compensation (5). Moreover, underlining the important role of functional imaging to capture the neural basis of compensatory processes, they describe how individual theories embed hyperactivity of regions commonly engaged during a task, on the one hand, and the recruitment of alternative areas or networks, on the other hand.

Resulting from the observation that older adults seem to recruit symmetric brain areas more extensively when compared to younger adults showing more asymmetric patterns, the hemispheric asymmetry reduction in older adults (HAROLD) model was developed (6). It mainly pertains to lateralization reduction of prefrontal cortex activity and the author sought to integrate findings from declarative and working memory (WM), as well as perception and inhibition. In more recent accounts (4), HAROLD was found to be consistent with explicit criteria of compensation, which we will describe in detail below.

Another intriguing finding of potentially compensating brain activity in aging is the overall increased recruitment of (pre-)frontal areas, while activity in occipital cortex is reduced. Following these results first noticed in studies of perception, attention, and recognition, the posterior–anterior shift in aging (PASA) model was advanced (7, 8), which includes criteria for compensation, as well (4).

While HAROLD and PASA emphasize on increased prefrontal cortex activation, other theoretical concepts define trajectories of compensatory mechanisms across the age span irrespective of a specific location. A case in point is the compensation-related utilization of neural circuits hypothesis [CRUNCH; (9)], which emerged from age-related hyperactivity. CRUNCH describes progressing over-recruitment of brain areas usually involved in the respective cognitive function across the life span. While there was and still is a debate as to whether over-activation of the HAROLD and PASA kind constitutes a compensation or rather a dedifferentiation-related phenomenon (6), CRUNCH is more explicitly viewed as a concept of compensation with implications for interventions to sustain compensation (10, 11). According to CRUNCH, hyperactivity in older adults happens “sooner,” i.e., at lower task demands, compared to younger adults. Correspondingly, the maximum of the load-activation function is reached at a lower cognitive load level, followed by a breakdown in cognitive performance, as well as in activity. Thus, the aging brain shows plasticity in recruiting additional neural resources to sustain cognitive functioning, but will inevitably fall short to do so beyond a specific resource ceiling (9).

One of the most recent and flexible accounts of compensation (5) is the scaffolding theory of aging and cognition [STAC; (12)]. Structural as well as functional alterations due to brain aging are suggested to challenge cognitive functioning. The impact of such challenges is compensated by scaffolding, which subsumes various processes of plasticity, including neurogenesis and the HAROLD and PASA patterns. Additionally, scaffolding can be enhanced by interventions such as cognitive training, see e.g., recent work by Goh and Park (13). The brain’s ability to scaffold in large parts subsumes all above-mentioned compensation models.

A different overarching perspective on compensation, explicitly including neurodegenerative disease in addition to normal brain aging, is expressed in the cognitive reserve (CR) framework (14, 15). CR is defined as “differences in cognitive processes as a function of lifetime intellectual abilities and other environmental factors that explain differential susceptibility to functional impairment in the presence of pathology or other neurological insult” (5). Concerning the translation of CR to the brain, there are passive models of CR (16), merely accounting for, e.g., difference in brain size or number of neurons. In addition, active models of CR refer to the brain’s reactions when facing cognitive challenges. Among such active models, so-called “neural reserve” (NR) accounts for the increased recruitment of usually task-positive networks by impaired individuals (15). Another active account of CR is “neural compensation” (NC), describing the recruitment of additional, alternative regions, or networks to sustain performance (14, 15). It has been shown that CR is more strongly correlated with regional fMRI activity than regional volumes (17), suggesting fMRI to be an ideal tool for investigating CR. Several reviews [see e.g., Ref. (18)] focused on evidence for neural correlates of CR in aging and there is another recent account of CR-related evidence in Alzheimer’s disease (AD) (19). There are several qualitative and quantitative reviews suggesting that all above-mentioned models of compensation are suitable to account for the brain’s reactions when faced with cognitive challenge at older age (20–22).

Despite the common interpretation of increased brain activity when facing cognitive challenges as compensatory, Grady (20), following up on an earlier review (3), stresses a prominent gap of knowledge across studies: in the majority of reports, authors claim activity to be compensatory without explicitly relating it to successful task performance. This is problematic, as the presence of hyperactivity could be due to unspecific effects unrelated to the cognitive function under investigation or a sign of dedifferentiation (6, 23). Moreover, Price and Friston point out that a compensatory interpretation of increased brain activity is inappropriate if one cannot ensure that the “impaired” group under investigation used the same cognitive strategies to perform a task compared to a control group (24, 25). Therefore, it is a necessary prerequisite to check for behavioral differences between groups, e.g., regarding accuracy or response latencies. If these measures do not differ between groups, activity differences can be interpreted in the light of compensation. In addition, Grady (20) concludes that the inclusion of task performance should be mandatory when interpreting a result as compensatory. Grady recommends terms such as “attempted” and “successful” compensation as suggested by Dennis and Cabeza (4) to categorize imaging findings of increased activation in aging or neurodegeneration to resolve discrepancies in previous reports. In the remainder of the introduction, we will describe the unifying account of compensation by Dennis and Cabeza (4).

### Criteria of attempted and successful compensation

**Figure 1:**
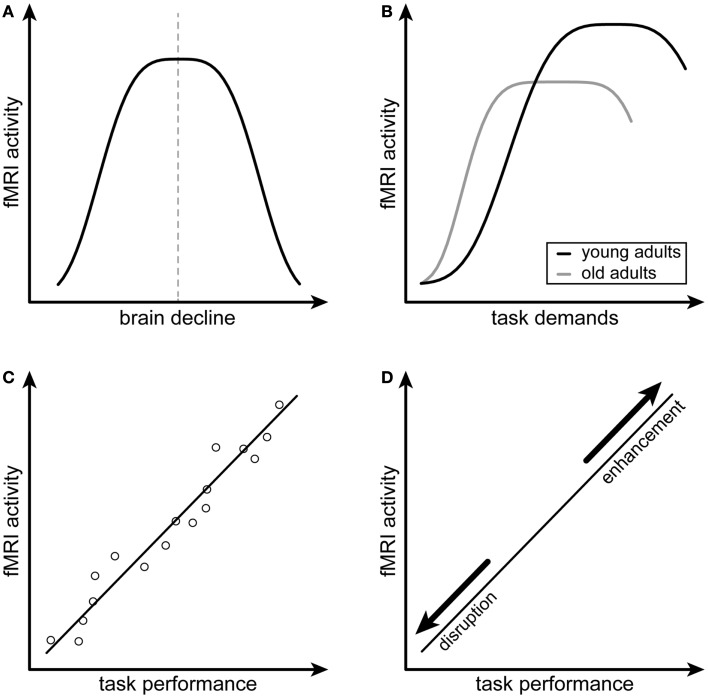
**Schematic illustration of the compensation criteria by Dennis and Cabeza (4)**. **(A,B)** depict the first and second criteria of attempted compensation, namely the inverted *U*-shaped relations of fMRI activity and neural decline as well as fMRI activity and task demands, respectively. **(C,D)** depict the first and second criteria of successful compensation, namely a positive correlation between fMRI activity and task performance as well as the alteration of this relationship after disruption or enhancement of the compensating brain region. **(A,B)** are adapted from Figure 37-3, p. 635, of the chapter by Dennis and Cabeza (4).

Dennis and Cabeza (4) try to resolve contradictory interpretations of increased activity, as well as different terminology regarding compensation, by first suggesting an overarching model and then by postulating specific criteria to assess potential compensation (Figure 1). Comparable to other models, their starting point are receding overall “neural resources” (e.g., number of neurons and their wiring) with advancing age. Following these dwindling neural resources caused by brain aging, neural supply to fulfill task requirements will be limited. The authors assume a mismatch between task demands and available cognitive processing resources, which justifies the need for compensatory hyperactivity. Ensuing from the existing ambiguity as to whether identified hyperactivity should be termed “compensatory,” the authors distinguish “attempted” and “successful” compensation. They further suggest two criteria for attempted as well as two for successful compensation, allowing to judge past and forthcoming work along these lines.

The first criterion for attempted compensation according to Dennis and Cabeza (4) is an inverted *U*-shaped relationship between increasing brain activity and structural brain decline (Figure 1A). Assuming such a relationship, activity should be highest when neural resources subside. Furthermore, there is strong evidence that a reversal point in this relationship exists, when the burden of brain decline is too high and attempted compensation will fail, yielding an inverted *U*-shaped curve [see e.g., Ref. (26)]. Second, attempted compensation is characterized by another inverted *U*-shaped relationship with task demands (Figure 1B). Resulting from a mismatch between available cognitive resources and task demands, activity should rise when the task becomes more complicated as manipulated by, e.g., increased cognitive load level, until it reaches a plateau. The rise and plateau of the curve will be reached at lower task demands at older age or in neurodegenerative disease, compatible with the CRUNCH (9) and the NR model (15). Similarly to the first criterion, a task can become too demanding given limited capacity of neural resources and cognitive processing at a certain point. This is when the curve will start to drift downwards, again yielding an inverted *U*-shaped relationship.

When the attempt to equate a lack of neural resources is positively related to better cognitive performance, the authors speak of “successful” compensation. Compared to attempted compensation, the concept of successful compensation thus goes further by requiring direct links with task performance. First, increased activity or connectivity should be positively correlated with task performance as measured by, e.g., reaction times or percent correct responses (Figure 1C). The second criterion for successful compensation concerns the manipulability of a potentially compensating region and resulting influence on task performance (Figure 1D). For compensation to be termed successful, the temporary disruption of a specific region by, e.g., transcranial magnetic stimulation (TMS) or transcranial direct current stimulation (tDCS) should yield a decline in performance.

Following the suggested criteria for attempted and successful compensation, Dennis and Cabeza (4) reviewed evidence regarding prefrontal cortex hyperactivity in aging and concluded that most studies revealed hyperactivity patterns fulfilling their criteria. Nevertheless, the second criterion of successful compensation, requiring a disruption of functioning in the respective area and a consequently declining performance, was clearly underrepresented in studies of healthy aging, as few studies using stimulation with, e.g., TMS and tDCS exist in the context of compensation. Additionally, the investigation of the entire *U*-shape for both criteria of attempted compensation is often not possible, as this would require the investigation of a very large age span. Furthermore, they concluded that PASA- and HAROLD-like patterns of hyperactivity could be subsumed under the proposed compensation model (4). As already mentioned above, an earlier recruitment of additional neural resources in aging as postulated in CRUNCH (9) is well compatible with attempted compensation. In addition to Dennis’ and Cabeza’s (4) extensive review, we would like to emphasize that STAC can be integrated in their compensation model as well, as the model comprises high flexibility regarding the actual form of scaffolding. Furthermore, NR and NC, characterizing the increased recruitment of usually task-positive networks versus the recruitment of alternative networks (5, 15) can likewise be accommodated in the suggested compensation framework, as Dennis and Cabeza include main as well as reserve neural resources in their model. In contrast, their model does not explicitly include a concept such as CR (14), hypothesized to be protective when facing neuronal decline across the life span. This is unproblematic for the current review, as the majority of studies on compensation in preclinical and early manifest neurodegeneration, which constitute our scope, do not directly incorporate an estimate of CR.

### Objectives of review

As mentioned above, we will use criteria suggested by Dennis and Cabeza (4) as a proxy to judge systematically whether models of compensation originally postulated for healthy aging can be applied to findings from preclinical and early manifest neurodegeneration, as well and if there is evidence for attempted and successful compensation in neurodegeneration. The application of these criteria to neurodegenerative disease is warranted in our opinion, as neuropathological processes that increase the need for compensation accumulate in the aging as well as in the diseased brain [see, e.g., Ref. (27, 28)]. Concerning brain structure, both healthy older and cognitively impaired adults suffer from neuronal loss, albeit with different trajectories (29–33). Correspondingly, Prvulovic and colleagues (21) advocate an integrative view of compensation for aging and neurodegeneration related brain changes as changes in functional activation patterns seems to follow the same rules in aging and dementia.

In addition, we will assess overlaps in compensating brain regions in two neurodegenerative disorders of interest, namely preclinical HD and aMCI. Finally, we will discuss implications of successful compensation for tailored interventions to sustain cognitive performance and at best delay disease onset.

## Recent Accounts of Compensation in Preclinical and Early Manifest Neurodegeneration

In the context of healthy aging, a vast amount of task-related as well as task-free fMRI studies exist that investigate the nature of altered activity and connectivity in healthy elderly [for reviews, see e.g., Ref. (9, 12, 18, 20–22, 34)]. In neurodegenerative disorders, evidence for activation patterns similar to healthy aging begins to accumulate (35). Especially, the preclinical and early manifest stages of these disorders constitute windows of opportunity for successfully sustaining compensation with different kinds of interventions (36–38). Therefore, the current review targets two disorders representing specific phases of neurodegeneration, namely preclinical HD and aMCI.

Huntington’s disease is a genetically caused hereditary neurodegenerative disorder. The genetic mutation irrevocably leading to manifestation of the disease can be tested for The Huntington’s Disease Collaborative Research Group (39), raising the possibility of identifying gene carriers decades before symptom onset. Gene carriers lacking overt motor symptoms are described as “preclinical” or “preHD,” while their estimated years to clinical onset can be calculated using a parametric survival model (40, 41). The unequivocal determination of the disease by gene status at a very early-stage renders HD, an ideal model to study preclinical neurodegeneration (42). Clinical onset of HD is characterized by the presence of motor symptoms (43), while cognitive decline and psychiatric disturbances precede overt motoric signs by several years [see e.g., Ref. (44–51)]. Compensation has been studied intensively in animal models of HD (52). The limited number of available studies in humans has been reviewed recently (47) but without applying the compensation criteria by Dennis and Cabeza (4).

Patients diagnosed with amnestic MCI are prone to convert to manifest Alzheimer’s disease (53, 54) and constitute the most studied subtype of MCI before the revised definition of prodromal AD was published (55–59). They exhibit cognitive decline in single or multiple memory domains prior to potentially converting to AD, with declarative and WM being the first domains to be affected (60). Relevant to task fMRI, patients with aMCI are still able to perform cognitively demanding tasks in the scanner environment. Recently, fMRI was established as a sufficiently reliable marker to monitor disease progression in aMCI, and fMRI test–retest reliability seems to be comparable to reproducibility in healthy elderly (61, 62). Reassuringly, this implies that compensatory mechanisms accompanying the disease should equally well be detected in healthy older adults.

### Compensation in preclinical neurodegeneration: Huntington’s disease

First evidence of compensation in pre-symptomatic gene carriers comes from a time discrimination fMRI task (63). Dividing preHD patients in a “close to onset” and “far from onset” sub-groups and comparing them to healthy controls, the authors found hyperactivity of anterior cingulate cortex and preSMA in “far from onset” preHD compared to both the controls and the “close to onset” group, although neither group differed in their reaction times. This hyperactivity was suggested to compensate for neural decline in the basal ganglia, reflecting the inverted *U*-shaped relationship postulated for attempted compensation. Paulsen and colleagues (63) did not compute correlations with performance and the relatively low sample size of seven subjects per group are limitations of that study.

As subtle motor abnormalities progress during the preclinical stage of HD (51), simple motor tasks have been identified as suitable paradigms for PET and fMRI studies (64). In a finger sequence tapping task with manipulations of speed and complexity, Klöppel and colleagues (65) found differential patterns of activity in supplementary motor area (SMA) and superior parietal lobe (SPL) in a group of preHD patients relatively far from onset. With regard to caudal SMA, the preHD group displayed increased activity across all task conditions compared to controls. Moreover, activity of caudal SMA was positively related to estimated years to onset, mirroring stronger attempted compensation with increasing neural decline. In contrast, preSMA activity diminished when approaching disease onset, pointing to differential processes in subregions of SMA. In right SPL, preHD showed hyperactivity in all but the most complex task condition, presumably reflecting the inverted *U*-shaped relationship with task demands in attempted compensation. Adding to these indices of attempted compensation, Scheller and colleagues (66) found a positive correlation of task-related effective connectivity from dorsal premotor cortex (PMd) to SPL with years to estimated disease onset in a re-analysis of the same dataset. This finding points to a potential *U*-shape regarding increased connectivity with increased neural decline, and therefore strengthens the assumption of attempted compensation.

## Working Memory and Compensatory Brain Activity in preHD

The most studied cognitive domain regarding compensation in preHD is WM, though studies differ concerning type of WM task, sample size, and years to onset of preHD participants. Indices of attempted compensation can be derived from functional connectivity analyses (67), which revealed a decline of left DLPFC connectivity only at high WM load levels in a modified Sternberg paradigm. In a longitudinal WM study from the same group (68), Wolf and colleagues did not find hyperactivity in the preHD group but hypoactivity of left DLPFC compared to controls at equal performance, which remained stable over 2 years. As their preHD participants were about 20 years from clinical onset on average, the finding suggests that a need for compensation might arise in a later stage of the disease, which was not captured in the investigated participants. Considering the right side of the inverted *U*-shaped relation of neural decline and compensatory activity, manifest HD participants showed declining WM performance accompanied by hypoactivity of the WM network, which increased with WM load (69). Concentrating on task-related deactivation, i.e., the “task-negative network,” Wolf and colleagues (70) conducted a longitudinal study of WM function in far from onset preHD across 2 years. Over time, higher functional connectivity of left anterior prefrontal cortex in preHD compared to controls remained stable. In contrast, connectivity in dorsal cingulate cortex increased in preHD, which was negatively correlated with motor functioning as a marker of disease progression. Taken together, task-negative regions typically display a compensatory increase in connectivity even in far from onset preHD participants, though compensation cannot be termed “successful,” as associations of this increase in connectivity with performance were not reported (70).

More recently, WM has been studied longitudinally in preHD and early manifest HD using a visuospatial n-back task (71) in the IMAGE-HD study (72–74). Cross-sectionally, Georgiou-Karistianis and colleagues found overall less activity in the WM network in preHD and early HD compared to controls, which further decreased with higher WM load, contradicting a compensatory reaction of core task-relevant regions (73). In a time-course analysis of the BOLD response, they found that in low WM load early HD seemed to recruit DLPFC more strongly than preHD and controls at equal performance, which can be interpreted as attempted compensation. The 18-months longitudinal data (72) show a very different picture of compensation, especially in preHD. When compared to baseline, hyperactivity in preHD was identified in prefrontal, medial frontal, precentral, and temporal regions, as well as in caudate and putamen. Moreover, such over-recruitment was not present in early HD and controls, reflecting the inverted *U*-shaped relation of hyperactivity and neural decline in attempted compensation (72). Of note, longitudinal over-recruitment of precentral gyrus and putamen was positively correlated with reaction time during the WM task, which suggests unsuccessful compensation, as hyperactivity was associated with slower response. The authors further observed reductions in cortico-cortical as well as cortico-subcortical functional connectivity in preHD, interpreted as a sign of network dysfunction potentially compensated by hyperactivity (72). Looking at the 30-month longitudinal data (74), the decline in functional connectivity between DLPFC and caudate still increased longitudinally. Conversely, patterns of hyperactivity in the above-mentioned regions plus inferior parietal lobe were again present in preHD in high WM load, confirming attempted compensation. Unfortunately, the authors did not report correlations with performance, preventing an interpretation concerning successful compensation.

## Further Cognitive Domains and Compensatory Brain Activity in preHD

Apart from WM, there is recent evidence on increased frontal activity in preHD across different cognitive domains. In a shifting response-set task targeting prefrontal cortex function, also measured as part of the IMAGE-HD study, Gray and colleagues (75) identified widespread hyperactivity in early HD and to a lesser extent in preHD compared to controls. Over-recruited regions in preHD included left inferior and superior frontal gyrus, left anterior insula, and precentral gyrus, as well as left caudate head, anterior putamen, and anterior pallidum. Apart from these indices of attempted compensation, the authors found preserved executive functions as well as task accuracy to be positively associated with DLPFC activity in early HD, but not in preHD (75). Thus, successful compensation seemed to be present in early HD but not in preHD, potentially reflecting a lack of need to compensate in the shifting response-set task in this relatively far from onset group (mean years to onset = 14.8 years). The importance of taking mean years to onset into account when interpreting compensation results is further underlined in a study investigating alertness (76), identifying hypoactivation in fronto-striatal networks in preHD close to onset, i.e., 10.8 years on average, compared to controls and far from onset preHD, approximately 33 years from onset. There is no definite convention in the field on the ascription of “far” and “close” to onset, which emphasizes the absolute necessity to report the exact measure of years to onset to enable the comparison of studies.

Interestingly, Wolf and colleagues (77) re-analyzed the same dataset with an emphasis on default-mode network (78, 79) changes in preHD, without dividing preHD participants in close to and far from onset. Taking two task-negative default-mode subsystems into account, preHD seemed to have lower within-subsystem connectivity, while between-subsystem connectivity was increased compared to controls, interpretable as attempted compensation. Moreover, lower connectivity in the left inferior parietal lobe was associated with shorter reaction times, which was interpreted as a mechanism to sustain performance by recruiting this usually task-negative region to a greater extent (77), pointing to successful compensation. Obtaining such a wealth of results with one dataset by employing different analysis strategies makes decisions on compensation challenging on the one hand, but on the other leaves the door open to beneficial re-analysis of datasets, especially when confirmation of successful compensation was missing.

Finally, specific frontal areas, namely superior and middle frontal gyrus, were further associated with compensatory responses in preHD in an emotion recognition task (80), accompanied by hypoactivation in an extensive network across three different emotion conditions. This additional recruitment of superior and middle frontal gyrus was positively associated with CAG repeat numbers, representing another measure of disease load, rendering the interpretation of frontal recruitment as compensatory more credible (80). Interestingly, medial frontal areas also form part of the DMN, mirroring the above-mentioned interpretation of sustained performance through supporting task-negative regions by Wolf and colleagues (77).

### Compensation in early manifest neurodegeneration: Amnestic MCI

One of the first cognitive functions to be impaired in MCI is declarative memory (60, 81). From structural as well as functional imaging studies, it is known that hippocampus and adjacent medial temporal lobe (MTL) regions subserve episodic memory and therefore play a key role in the progression of MCI to manifest dementia (82–84). Therefore, we will emphasize on MTL-related compensation reports in the following paragraphs. As there are two reviews (85, 86) summarizing episodic memory findings in MCI relating to MTL, we will emphasize on their conclusions. In addition, we will provide a more detailed account of WM studies in aMCI (87), as WM is affected in during the course of the disease as well. Finally, we will summarize reports regarding other cognitive functions fulfilling at least one criterion of successful compensation.

## Compensation in Episodic Memory and Medial Temporal Lobe Activity in aMCI

In the first of two qualitative reviews on MTL function, Dickerson and Sperling (85) describe hypo- as well as hyperactivity in the declarative memory network across different stages of neurodegeneration. In early MCI, hyperactivity of MTL, as opposed to hypoactivity in late MCI and mild AD, shows the inverted *U*-shaped relationship of neuronal decline and compensatory activity. Building on these observations in a second review, Sperling and colleagues (86) embed MTL-related findings in the context of the default-mode network and elaborate on the interplay of MTL with other regions that form parts of DMN, e.g., the precuneus, and their role in memory functioning. They conclude that it remains unclear if the increase in MTL activity is compensatory or a sign of inefficiency or neuronal demise.

Several studies discussed in the review pointed to an inverted *U*-shaped relationship of MTL activity and disease progression (88–90). Miller and colleagues (90) found over-recruitment of MTL during scene encoding to be exclusively predictive of future cognitive decline. In addition to MTL, hyperactivity of the cortical attention network was present in MCI and mild AD (88). Furthermore, memory load has been found to regulate MTL activity in aMCI compared to controls, which might characterize the right side of the inverted *U*-shape with a breakdown of attempted compensation when task demands increase (91). In addition to these reports of attempted compensation, Heun and colleagues (92) specifically associated hyperactivity with successful trials. In their work, increased activity in different areas of bilateral prefrontal cortex was identified for hit and correct rejection responses in MCI patients, but not in controls. As both response categories relate to successful performance of the task, the study comes closest to fulfilling the first criterion for successful compensation, though the authors did not explicitly correlate a measure of performance and BOLD response in respective regions (92). Correspondingly, hypermetabolism in DLPFC has been identified in a PET study of highly educated aMCI patients that later converted to AD, while the same patients exhibited hypometabolism of MTL (93).

Despite a considerable amount of studies linking MTL activation in episodic memory to compensatory mechanisms, there is convincing evidence for the interpretation of such hyperactivity in terms of inefficiency as well. In a recent study, MTL hyperactivity was interpreted as a correlate of encoding dysfunction (94). This interpretation was expanded by Bakker and colleagues (95), who pharmacologically manipulated hippocampal hyperactivity. Participants whose hyper-recruitment was reduced by applying an antiepileptic drug showed better cognitive performance, clearly arguing against successful compensation, which would require a positive relation of performance and hyperactivity. Moreover, MTL activity during encoding correlated with correct recognition in healthy controls but not in aMCI patients, objecting to successful compensation (96).

Intriguingly, when analyzing large-scale brain networks instead of focusing on MTL exclusively, a follow-up analysis of the same data by Protzner and colleagues (97) revealed that activity in a different network including inferior temporal gyrus was related to performance in aMCI participants, potentially compensating for deficient MTL functioning. In a different episodic memory encoding and retrieval task, Clément and colleagues (98) found increased left ventrolateral prefrontal activation during the encoding phase, which positively correlated with successful retrieval in aMCI patients. This evidence again points toward successful compensation of deficient MTL functioning [but see conflicting results in Ref. (99)]. Recently, Parra and colleagues (100) identified activation patterns consistent with both attempted and successful compensation in an emotional memory fMRI task with a post-scan recognition test. Activity in MTL structures, namely hippocampus and parahippocampal gyrus, followed an inverted *U*-shape across healthy controls, MCI, and mild AD patients. Moreover, MTL activity was positively correlated with recognition performance in all groups.

Latest evidence from a quantitative activation likelihood estimation [ALE; see e.g., Ref. (101)] meta-analysis integrating above-discussed and further studies [e.g., Ref. (102, 103)] of episodic memory in preclinical and manifest AD, suggests a differential course of activity across disease progression in several anterior hippocampal subfields (104). As previous studies may sometimes have failed to differentiate encoding and retrieval and did not investigate hippocampal subfields in a fine-grained fashion, these different hippocampal activity trajectories may help to resolve above-mentioned contradictory conclusions on MTL activity in aMCI to a certain extent. Moreover, precuneus, often reported together with MTL, seems to be increasingly activated in encoding tasks in mild AD, but not yet in MCI. To unambiguously determine if MTL or other areas, such as DLPFC (92, 93, 102, 103) or precuneus (105), successfully compensate to sustain episodic memory in aMCI, studies investigating broader ranges of neurodegeneration with increased sample sizes and longitudinal observations (106) are necessary. Taken together, there is evidence of attempted as well as successful compensation of episodic memory function in aMCI.

## Compensation in Working Memory in aMCI

As WM function is often impaired in aMCI (107, 108) and might be predictive for conversion to AD (109), it has been investigated in task fMRI studies during the last years. One of the first indications of attempted compensation in WM was revealed by Yetkin and colleagues (110), who investigated small samples of healthy elderly, aMCI, and mild AD patients in a simple visual WM task, in which groups reached equal performance. Applying rather liberal statistical thresholds, they found frontal and temporal areas to be hyperactivated in aMCI and mild AD compared to controls, while AD patients showed reduced activity compared to aMCI.

To investigate whether emotional content differentially affects WM performance, Döhnel and colleagues (111) used an n-back task with emotional stimuli. Better WM performance within aMCI specifically for negative emotional content was accompanied by increasing activity in right precuneus as compared to control subjects. Interestingly, right precuneus was found to be more activated in aMCI compared to controls at low WM load in a different study (112). Moreover, at higher WM load, activity in the same region was reduced together with greater deactivation of a nearby cluster in medial precuneus, fitting an inverted *U*-shaped relationship of activity, and task demands required for attempted compensation. Kochan and colleagues interpreted this sensitivity of precuneus to WM load manipulations in the context of potential biomarkers of disease progression and showed that greater deactivation in posteromedial cortex predicted decline in everyday functioning 2 years later (113). The involvement of default-mode network regions in aMCI was followed up on by Migo and colleagues (114), who found relative hyperactivity in the sense of reduced task-related deactivations in right insula in a verbal N-back task, which were also load-dependent, indicating attempted compensation. Such default-mode network changes are mirrored by studies in preHD (77, 80), pointing toward a similarity of compensation across disorders.

Further evidence for attempted and successful compensation comes from a verbal delay match to sample WM task (115), though aMCI and control samples were rather small (*n* = 8, respectively). They found hyperactivity of several regions in aMCI during encoding and maintenance and additionally correlated activity with response time. In the encoding phase of the task, activity in right inferior occipital cortex and MTL was positively correlated with performance, while the same was true for left middle frontal gyrus and left precentral gyrus during maintenance. During recall, a positive relation was found in left hippocampus, reflecting MTL involvement in successful WM performance [see e.g., Ref. (116–118)] and further paralleling declarative memory findings discussed above. Overall, the authors observed differential correlation patterns in HC and aMCI, suggesting that alternative neural resources subserve intact WM functioning in aMCI.

Inducing high WM load with a complex WM span task suggested to be predictive for conversion to AD (109), Faraco and colleagues (119) found hyperactivity of frontal, parietal, as well as medial, and lateral temporal lobe regions. These activations are consistent with previous studies on WM [e.g., Ref. (112, 115)] and in parts on episodic memory (86), though the authors did not restrict their MCI group to aMCI. This pattern of hyperactivity consistent with attempted compensation was accompanied by overall greater dispersion of activity compared to controls, supporting the HAROLD account of compensation (6). Again, the authors observed an overlap of potentially compensating regions, namely DLPFC and MTL, to those found in episodic memory studies (see above). Furthermore, activity in superior temporal gyrus, inferior frontal gyrus, and Heschl’s gyrus was positively related to hit rate in the short term memory task, corresponding to the first criterion of successful compensation (119).

Recently, compensation-related activity in WM has been investigated by directly enhancing cognitive functioning with caffeine in a double-blind placebo-controlled study using a verbal 2-back task (120). Comparing aMCI patients to controls, caffeine-related activity was increased in posteromedial cortex, inferior parietal cortex, occipital cortex as well as different subregions of MTL, contrasting pronounced recruitment of frontal areas in controls, compatible with PASA. Moreover, functional connectivity of the well-established WM network (121, 122) was increased in aMCI, but not in controls. These results suggest that regions previously found in an attempted compensation context might be candidates for enhancing interventions. Nevertheless, the authors did not explicitly correlate performance and activity, which leaves the question of successful compensation unanswered (120).

## Evidence of Successful Compensation in aMCI Across Different Cognitive Domains

Attempted compensation criteria were fulfilled in studies of episodic memory and WM (see above). Unfortunately, compensation has less extensively been assessed in other domains [see e.g., Ref. (123)], hence, we restricted the preceding paragraphs to episodic and WM. As it proved more difficult to find accounts of successful compensation, we will summarize evidence of successful compensation across different cognitive domains. Reviewing these domains separately would not be useful due to the limited number of studies. We will mainly focus on the first criterion of successful compensation demanding a positive correlation of task performance and hyperactivity, as studies on the second criterion, requiring disturbance of compensatory areas, scarcely exist.

Investigating single domain aMCI across four fMRI tasks examining language comprehension, visuospatial attention, episodic memory and empathy, Lenzi and colleagues (124) identified attempted compensation patterns in the sense of increased activity in aMCI compared to controls in all domains but empathy. Furthermore, the authors correlated this hyperactivity with neuropsychological scores quantifying performance in the respective domains. Interestingly, Lenzi and colleagues found negative correlations of hyperactivity and neuropsychological performance in the language comprehension, as well as episodic memory domains. This contradicts successful compensation, though correlations did not directly incorporate within-scanner task performance. Furthermore, they found a positive correlation of right inferior frontal gyrus hyperactivity and neuropsychological scores (i.e., Trail Making Test A) in the visuospatial attention domain (124). This finding comes closest to successful compensation, though as already mentioned above; the neuropsychological test does not constitute a direct measure of task performance.

Focusing on parietal lobe function in aMCI, Jacobs and colleagues (125) studied a mental rotation task in aMCI patients and matched healthy controls. They indeed observed higher parietal activation in aMCI at a high load level, while both groups performed equally well. Investigating this parietal hyperactivity further in a Granger causality analysis, they found that increased connectivity of left parietal lobe with posterior cingulate cortex, middle occipital cortex, and inferior parietal cortex in aMCI correlated positively with performance, pointing to successfully compensating by increasing connectivity between these areas (125). Besides identifying successful compensation, this work reflects the transferability of the suggested framework from pure activity to functional and effective connectivity studies (4), broadening the scope toward network-related interpretations.

In another study focusing on executive functions, Clément and colleagues (126) investigated stimulus manipulation as well as divided attention in two separate fMRI tasks, splitting their aMCI participants in two groups according to cognitive performance as in previous work (102, 103). The higher-cognition aMCI group exhibited hyperactivity compatible with attempted compensation compared to controls in both fMRI tasks, whereas the lower-cognition aMCI group failed to recruit additional areas. Moreover, during divided attention, fronto-striatal activity, namely in anterior cingulate cortex, left caudate and putamen, left insula, as well as left inferior frontal gyrus showed a positive relationship with task performance, fulfilling the first criterion of successful compensation (126). Restrictively, this correlation pertains to a group of 12 aMCI participants and we think that more evidence of successful compensation across various cognitive domains should be gathered in larger groups of aMCI patients.

We have summarized compensation-related accounts with an emphasis on episodic and WM in the preceding paragraphs. From the reviewed studies, it is evident that certain regions may be part of compensatory mechanism across different cognitive domains. Prefrontal regions such as dorsolateral prefrontal gyrus and inferior frontal gyrus were related to compensation in both episodic and WM. The same is true for MTL, with an emphasis on episodic memory. Moreover, prevalence of compensation in regions of the DMN as already observed in preHD, seems to pertain to aMCI, as well. Here, the precuneus was most often reported across studies as a promising candidate region and certainly warrants more in-depth investigation. It would be too far-fetched to subsume the mentioned regions in a common “compensatory network,” as large heterogeneity across studies has to be acknowledged, as well. Nevertheless the concept of certain regions concerting compensation across cognitive domains remains of great interest (127).

## Integration of Reviewed Studies

### Attempted and successful compensation in preclinical and early manifest neurodegeneration

Corresponding to evidence of healthy elderly’s intrinsic brain activity being more similar to AD patients’ than to healthy young adults’ (35), our overview of compensation-related studies in preHD and aMCI strongly suggests similar mechanisms of attempted and to a smaller extent of successful compensation in early neurodegeneration compared with healthy aging (4). The most extensively investigated cognitive domain was declarative memory in aMCI, with multiple accounts of attempted compensation.

The first criterion of attempted compensation, the inverted *U*-shaped relationship of hyperactivity and neural decline, was most often identified in the reviewed studies (85, 86, 88–90, 98, 111, 114, 115), especially by work not only including preHD and aMCI groups but also manifest HD and mild AD. This indicates that the assumed inverted *U*-curve typically covers a broad range of disease stages. The second criterion of attempted compensation, which is represented by an inverted *U*-shaped relationship of hyperactivity and increasing task demands, was less often fulfilled in the reviewed studies. In several accounts, both criteria of attempted compensation were observed together, see, e.g., Ref. (112, 114).

Regarding successful compensation, we faced a more mixed picture, mostly due to the lack of reported correlations of hyperactivity with performance as required in the first criterion of successful compensation, and missing intervention studies targeting the second criterion of successful compensation. More recent accounts, especially for aMCI, identified successful compensation (115, 119, 124–126), while evidence in preHD is virtually not available, owing to overall smaller numbers of compensation-related studies. Studies proving compensation through manipulation of the proposed mechanisms remain rare in healthy aging research, as well (4). Most authors report equal performance of healthy controls and preHD/aMCI, though explicit reports of positive associations between performance and hyperactivity are needed to render the assumption of compensation less speculative. Extending the model of attempted and successful compensation to early neurodegeneration, we further stress that the compensatory nature of correlations found in affected individuals should be verified by contrasting them with results obtained from healthy control groups. Such a contrast of positive activity-performance correlations in affected individuals to a non-significant correlation in healthy controls will help to underline a compensatory interpretation. Ideally, correlations with markers of disease load (e.g., estimated years to onset in preHD) can additionally be computed for regions exhibiting task-related hyperactivity [see e.g., Ref. (65, 66, 72, 80)]. A positive association of hyperactivity with disease load would further support the compensatory nature (c.f. first criterion of attempted compensation) and make it less likely that observed effects are a primary disease effect. Altogether, criteria of compensation derived from compensation models of healthy aging can very well be applied to early stages of neurodegeneration.

### Mutual patterns of compensation of preHD and aMCI in WM function?

The differential nature of cognitive decline in preHD and aMCI (48, 128, 129) led to an emphasis of studies on cognitive functions foremost disturbed in the respective disorder. WM has been investigated in both diseases and therefore allows a direct comparison. As apparent from Table 1, there is weak evidence for successful compensation in aMCI and none at all for preHD, which brings our discussion to focus on attempted compensation.

**Table 1:** **Summary of studies investigating working memory function in aMCI and preHD in chronological order**.

Study	Neurodegenerative disease	Attempted compensation		Successful compensation	
		*U*-shaped relation of neural decline and fMRI activity	*U*-shaped relation of task demands and fMRI activity	Positive correlation of performance and fMRI activity	Decline in performance when disrupting activity
Yetkin et al. (110)	aMCI/mild AD	+ (MCI and AD) right SFG, bilateral MTG, bilateral MFG, ACC, bilateral fusiform gyrus	n/a	n/a	n/a
Döhnel et al. (111)	aMCI	+ Right precuneus	n/a	n/a	n/a
Kochan et al. (112)	aMCI	+ Right ACC, right precuneus	+ Right ACC, right precuneus, PCC	n/a	n/a
Bokde et al. (115)	aMCI	+ rMTG, lSTG, precuneus, left fusiform gyrus, left lingual gyrus, PCC/ACC, left IFG, left MFG, right SFG, left caudate, right cerebellum	n/a	+ Right inf occipital, rMTG, lMFG, left precentral, left hippocampus	n/a
Faraco et al. (119)	MCI	+ Left Heschl’s gyrus, left amygdala, left planum temporale, left parietal operculum, left parahippocampus, left hippocampus, left insula	n/a	+ Heschl’s gyrus, IFG, STG	n/a
Migo et al. (114)	aMCI	+ Right insula, right lingual gyrus, hippocampus	+ Right insula, right lingual gyrus	n/a	n/a
Haller et al. (120)	aMCI	+ Bilateral temporal gyrus, bilateral parietal cortex	n/a	n/a	n/a

Wolf et al. (67)	PreHD	n/a	+ Left DLPFC	n/a	n/a
Wolf et al. (69)	Early HD	n/a	+ Left MFG, left IFG, right IPL, bilateral precuneus, left SPL, left putamen, right cerebellum	n/a	n/a
Wolf et al. (68)	preHD	–	n/a	n/a	n/a
Wolf et al. (70)	preHD	+ Left middle frontal gyrus, dorsal cingulate cortex	n/a	n/a	n/a
Georgiou-Karistianis et al. (73)^a^	preHD/early HD	–	+ (Early HD only), bilateral DLPFC	n/a	n/a
Georgiou-Karistianis et al. (72)	preHD/early HD	+ (preHD), bilateral DLPFC, left medial frontal cortex, ACC, right primary motor cortex, right temporal gyrus, right fusiform gyrus, left insula, right precentral gyrus, left putamen, left caudate	n/a	– (Unsuccessful)	n/a
Poudel et al. (74)	preHD/early HD	+ (preHD), left DLPFC, left MFG, bilateral caudate, bilateral putamen, PCC, left MTG	+ (preHD), left MFG, bilateral caudate, bilateral putamen, left PCC, left MTG	n/a	n/a

*^a^This study is reported in a non-chronological fashion, as it represents baseline results of the two following studies*.

*+, criterion fulfilled; –, criterion not fulfilled; n/a, not assessed*.

Similarly to WM-related compensation in healthy aging, compensating regions in preHD and aMCI were identified in prefrontal cortex, in both lateral and medial parts. Such frontal findings were present in four of seven studies in aMCI and six of seven studies in preHD (Table 1). Additionally, midline structures such as medial frontal cortex and precuneus were often identified, as were parts of temporal gyrus and insular cortex, all areas that form part of the default-mode network (78). Default-mode regions are usually task-negative across a wide variety of tasks, while they concomitantly exert task-sustaining functions (130). Interestingly, reduced task-related deactivations have been linked to healthy aging and neurodegeneration before (131–133). In a compensatory fashion, reduced deactivation leading to increased recruitment of DMN areas might sustain cognitive performance [see e.g., Ref. (77) for preHD and Ref. (114) for aMCI]. Specifically, precuneus activity, as reported in studies of episodic and WM in aMCI, might not only be part of the DMN, but may also be involved in concerting the interplay between DMN and task-positive networks (127). It therefore represents a promising compensation candidate and should be investigated further across cognitive domains and neurodegenerative disorders.

Unfortunately, statements about parallels in WM-related compensation remain speculative concerning successful compensation, as correlations of hyperactivity and performance are mostly missing (Table 1). Thus, we can only assume prefrontal task-positive as well as DMN-related task-negative regions to overlap in attempted compensation in both disorders. Overall, more studies investigating WM in both MCI and preHD are needed to decide on common compensating areas and to enable us to summarize such accounts more systematically.

### Methodological considerations and implications for future studies

As different models of compensation have been suggested over the last decade, it is not surprising to find high heterogeneity in studies aiming at investigating compensatory neural mechanisms. Moreover, sample size decreases when focusing on neurodegenerative disorders, which may lead to false negative, but also false positive findings, when reported effects are driven by only few cases (134–136). Such shortcomings can partly be resolved by subsuming studies in quantitative meta-analyses, as was done by Nellessen and colleagues (104) in the case of MTL in aMCI (104). Furthermore, larger multicenter studies capturing a broad range of neurodegeneration longitudinally (49, 54) may prevent such dissent in the future. Large samples will open the door to investigating preclinical and early neurodegenerative disorders as continua, rather than artificially dividing them into more and less severely impaired groups, providing the opportunity to follow compensatory trajectories along with neural decline.

More sophisticated statistical analysis approaches to disambiguate potential compensatory effects are needed as well. Moderating and mediating influences of different variables on compensatory hyperactivity could be investigated [see Ref. (5, 137) and, e.g., Ref. (138) for an exemplary account of moderation analysis]. In addition, path analyses have already been applied to verify CR accounts of compensation (139).

Moreover, modeling fMRI data to assess effective connectivity, e.g., with dynamic causal modeling (140) or Granger causality (141), will provide more mechanistic insight into compensatory mechanisms [see e.g., Ref. (66, 125)]. The interplay between nodes of a task-relevant network expressed by decreases and increases in effective connectivity to sustain task performance might especially be useful to identify targets for compensation-related interventions (142, 143).

As fMRI provides an indirect measure of neural activity by relying on the BOLD response, effects of neuropathology on neurovascular coupling might influence resulting brain activity, even to a greater extent in neurodegenerative disorders than in healthy aging (144). The hemodynamic response function is altered in certain cortical regions in these groups (145) and suggestions have been made to account for such influences (146). Furthermore, cognitive tasks may have a different impact on BOLD signal variability than motor tasks (147). To date, a way to account for confounding influences on the hemodynamic response function is to gather data on brain structure and vascular function together with the fMRI task. An exemplary account of controlling for such influences is the above-reviewed study by Haller and colleagues (120) including voxel-based morphometry, tract-based spatial statistics, and arterial spin labeling measures. Besides accounting for influences on the BOLD response, it is of upmost importance to carefully select the fMRI task with regards to cognitive abilities of the investigated patients (24). Ascertaining equal performance of impaired and control groups and in addition linking performance to increased activity, is a necessary prerequisite to unambiguously assess compensation (3, 20, 24).

As an interim conclusion, we propose certain cornerstones to design studies appropriate for the investigation of compensation following the criteria by Dennis and Cabeza (4): ideally, a sample to investigate compensation should cover a large range of preclinical and manifest disease stages to capture most of the inverted *U*-shaped curve relating neural decline to compensatory activity. Moreover, this could be complemented by longitudinal studies following the course of compensatory activity in the same individuals. Second, an experimental manipulation of task demands represented by, e.g., different cognitive load levels should be comprised in the design to investigate the second inverted *U*-shaped relation for attempted compensation. Moreover, including different load levels precludes ceiling effects, as variance in performance increases with difficulty, facilitating meaningful examination of correlations. Such correlations of task performance and increased activity should be computed in every study investigating compensation and be reported regardless of being negative, positive, or insignificant (see above). Concomitantly, finding a behavioral measure that is sensitive enough to detect compensation proves to be a great challenge with regards to task selection (47). An ideal measure should show neither floor nor ceiling effects and thus encompass enough variance to distinguish compensating individuals within the sample. As already stated above, we recommend correlating increased activity with markers of disease load if available, delineating compensatory recruitment along with increasing neuropathology. Finally, to fulfill the second criterion of successful compensation, study designs should include manipulations of potentially compensating regions by, e.g., TMS or tDCS, to observe disruption or enhancement of performance as a reaction to up- or down-regulation of the respective region. We are aware that the last criterion is most difficult to fulfill, as stimulation expertise has to be gained and compensating target regions for stimulation as well as suitable control regions have to be identified beforehand. Nevertheless, fulfilling this criterion leads to most unambiguous statements on compensation and outweighs simple correlation coefficients. Linking such compensation-oriented stimulation interventions with TMS or tDCS to current efforts on brain stimulation in neurodegenerative disease, we will provide a brief overview of these promising accounts in the following section.

### Implications for designing interventions

#### Non-invasive brain stimulation

Both preHD and aMCI might be target stages for interventions by stimulation or training to prolongate disease progression (36, 148). Therefore, it is of upmost importance to identify successfully compensating brain areas to sustain and possibly boost their function with the help of precisely tailored interventions (149). When successful compensation of a certain region is ensured, it will constitute an ideal intervention target. Such attempts, mainly in the prefrontal cortex in healthy elderly, have confirmed the HAROLD effect in bilateral DLPFC (150, 151), and in old adults with subjective memory impairment (152). Encouragingly, positive performance effects after rTMS have been observed in mild AD, as well (153). Existing evidence suggests that brain stimulation can enhance or sustain cognitive function to a certain extent [for a review, see Ref. (154, 155)]. When focusing on compensatory connectivity rather than activity, TMS further provides an ideal means of enhancing connections between areas sustaining performance (143, 156). Being less aversive for participants and easier to apply for the experimenter, first studies hint toward a beneficial effect of tDCS stimulation protocols on learning and memory in MCI (157); for a review, see Ref. (158).

#### Physical activity and cognitive training

A more indirect, purely non-invasive way to enhance compensation is represented by interventions, which are not localized to a specific brain area of interest. Such interventions often consist in physical activity or cognitive training programs. Physical activity seems to have a positive effect on cognition in intact older adults, as well as in MCI (159, 160). Additionally, interventions combining physical exercise and cognitive training seem to have a maintenance effect on cognitive function in HD patients (161, 162). Most studies on cognitive training interventions focus on healthy elderly and MCI or mild to moderate AD, with the idea to possibly delay onset of manifest dementia (163) by boosting compensation (164). There are several systematic literature reviews and meta-analyses on cognitive interventions in MCI (165–168). First promising accounts of monitoring training effects with fMRI exist (169), as well as attempts to foster WM function in aMCI (170). Parallel to the lack of systematic studies to investigate explicit criteria of compensation, there is an even bigger lack of studies systematically evaluating training interventions in large groups of patients with sufficient follow-up periods. Moreover, exercise and cognitive training interventions do not target a specific brain region. Observing their effect on established compensatory mechanisms, ideally measured by fMRI activity changes, may clarify if such mechanisms can be modified.

## Conclusion

In the current review, we sought to disambiguate the interpretation of over-recruitment of certain brain areas in early neurodegenerative disease as “true” compensation. To this end, we, presented criteria for attempted and successful compensation emerging from research on healthy elders (4) and reviewed recent compensation-related evidence from task fMRI studies on preclinical HD as well as aMCI according to these criteria. Having evaluated all studies regarding the above-mentioned criteria, we mostly identified attempted compensation in early neurodegeneration, while fewer studies contained evidence for successful compensation, due to missing reports of task performance correlations. Such a lack of evidence for successful compensation was most prominent in preHD. Furthermore, tentative parallels in compensatory mechanisms of working and episodic memory within aMCI, as well as in WM between aMCI and preHD were identified.

Due to the lack of studies fulfilling criteria of successful compensation, we suggest a study design following these criteria to unequivocally determine compensation in future studies. Therewith, evidence of recent intervention studies was discussed toward manipulation of successfully compensating regions by non-invasive brain stimulation or cognitive training. Through tailoring interventions to such compensating regions, we are confident that impact of interventions will be more precise, leading to overall increased efficacy. Overall, models of compensation along with explicit criteria of compensation from cognitive neuroscience of aging are suitable to characterize evidence from early neurodegeneration and to form a basis for future intervention studies.

## Conflict of Interest Statement

The authors receive funding from the CHDI foundation.
